# Supplementary material for: Modeling and Global Sensitivity Analysis of Strategies to Mitigate Covid-19 Transmission on a Structured College Campus
Source: Bull Math Biol. 2023 Jan 13;85(2):13. doi: 10.1007/s11538-022-01107-2 (PMC9837465; doi:10.1007/s11538-022-01107-2)
Supplement: Supplementary file 1 — (pdf 2804 KB) [file 11538_2022_1107_MOESM1_ESM.pdf]

Supplementary File 1: Modeling and Global Sensitivity Analysis of Strategies to Mitigate Covid-19 Transmission on a Structured College Campus

Lihong Zhao*, Fabian Santiago*, Erica M. Rutter, Shilpa Khatri, Suzanne Sindi†

A Variance and Global Sensitivity Analysis

The formulation for the spread of COVID-19 in a classroom, detailed in the main text, distinguishes between close (droplets) and far (aerosols) transmission (see Section 3.1 for details). In this document we present the variance and Sobol analysis results with a 10% chance of infection from 'far' contacts.

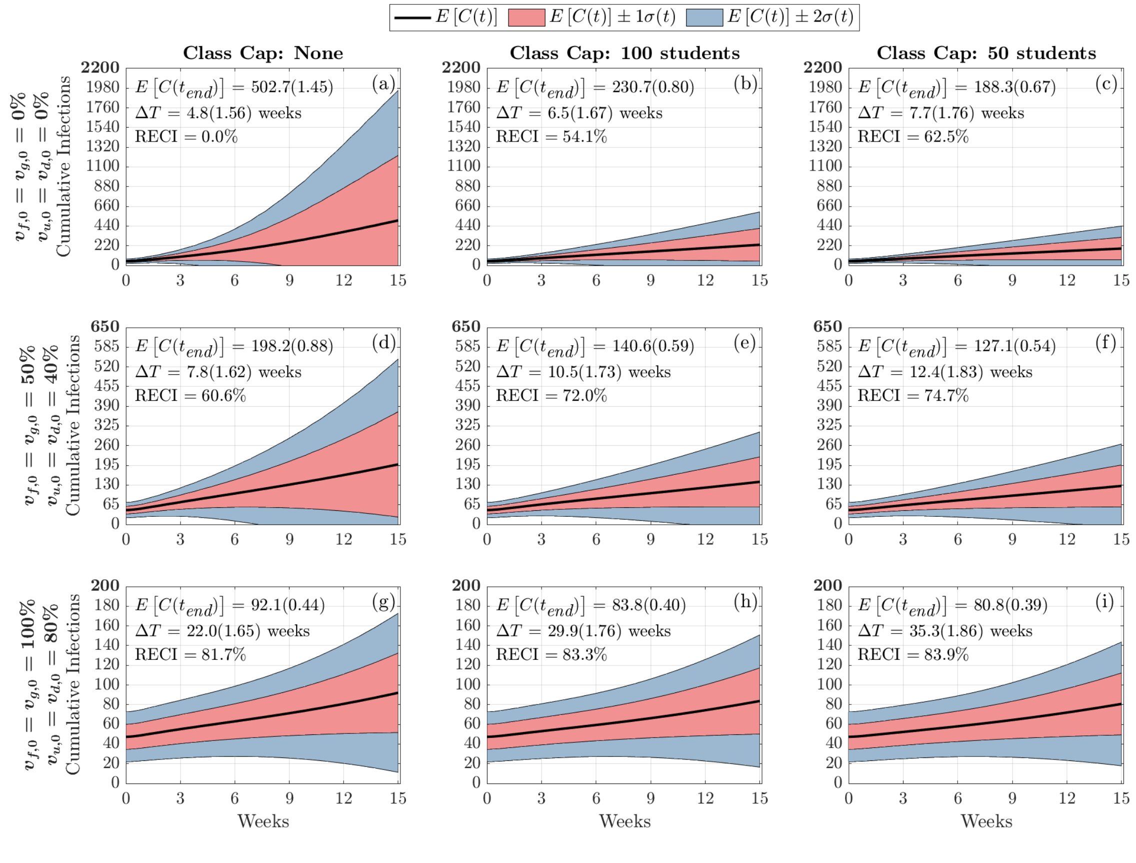

Fig. A1 Distribution of Cumulative Infections. Figures show the distribution of cumulative infections over the span of a semester (15 weeks) and we allow the contact, infection parameters, and initial number of infectious individuals to vary (See Table 1 in main text). The mean and coefficient of variation for the doubling time and total cumulative infections are reported in each subplot. The reduction in expected cumulative infections (RECI) from case (a) with no vaccination and no class caps is presented in each subsequent case.

*These authors contributed equally to this work.
†Department of Applied Mathematics, University of California, Merced. ssindi@ucmerced.edu

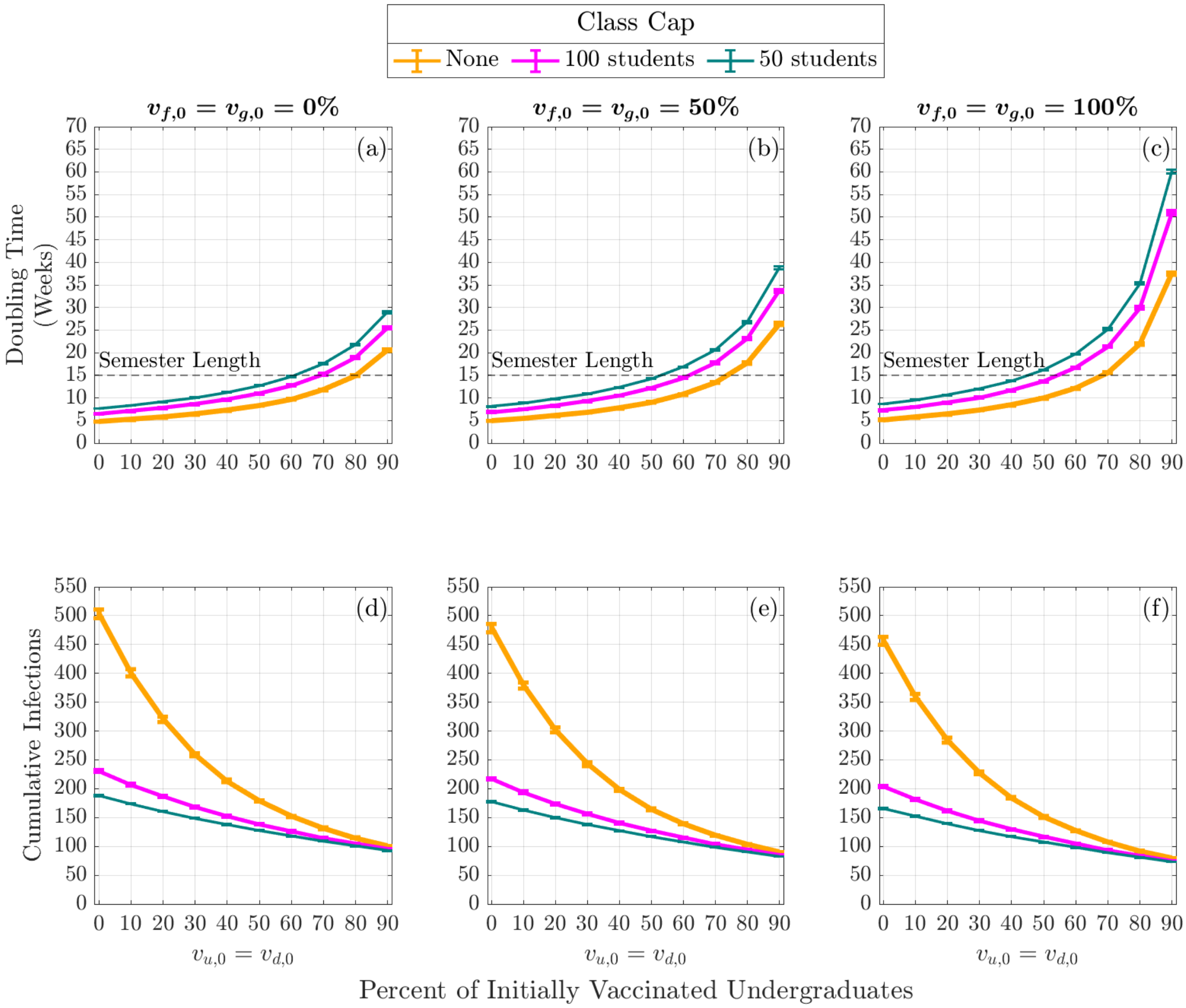

Fig. A2 Expected Infection Doubling Times and Cumulative Infections by Class Capacity and Percent of Vaccinated Undergraduates. The expected cumulative number of infection (cumulative infections) by the end of the semester and the expected doubling time computed during the first four weeks of the semester. The error bars are a 95% confidence interval.

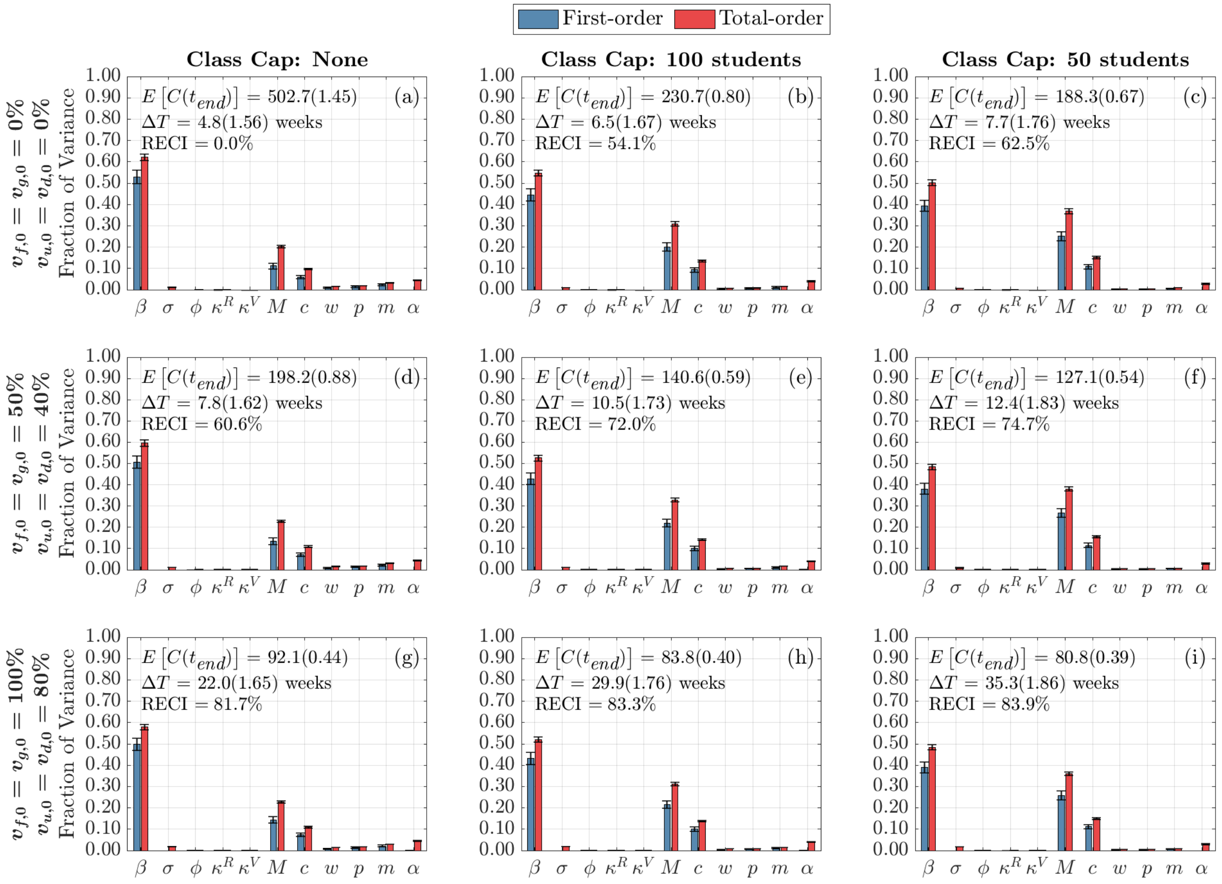

Fig. A3 Global Sensitivity Analysis of Infection and Contact Parameters on Infection Doubling Time. First-order (blue) and total-order (red) Sobol Indices are shown as well as the standard errors. The mean and coefficient of variation for the doubling time and total cumulative infections are reported in each subplot. Each column represents three class cap scenarios: none, 100 student, and 50 student caps. Each row represents one of three vaccination scenarios at the start of the semester. First row: 0% vaccination; second row: 50% of faculty, 50% graduate students, and 40% of under- graduate students vaccinated; third row: 100% of faculty, 100% graduate students, and 80% of undergraduate students vaccinated.

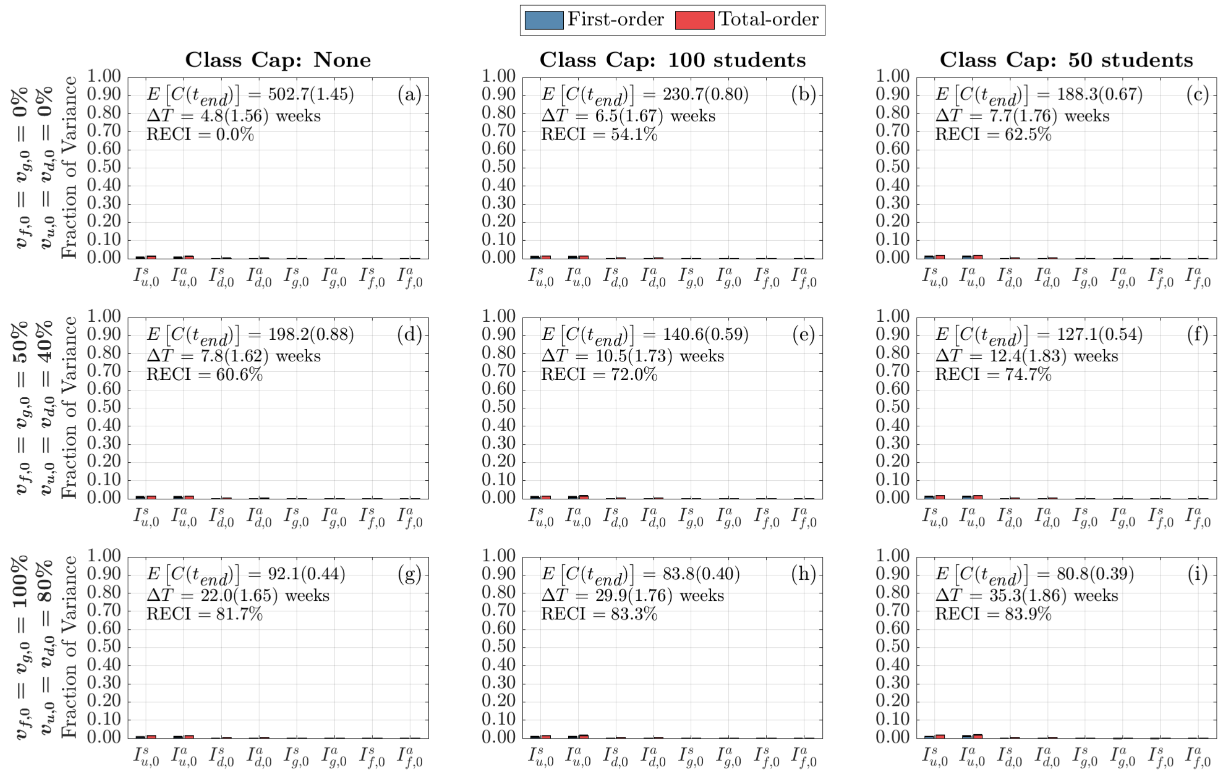

Fig. A4 Global Sensitivity Analysis of Doubling Time to Initial Conditions. First-order (blue) and total-order (red) Sobol Indices are shown as well as the standard errors. The mean and coefficient of variation for the doubling time and total cumulative infections are reported in each subplot. Each column represents three class cap scenarios: none, 100 student, and 50 student caps. Each row represents one of three vaccination scenarios at the start of the semester. First row: 0% vaccination; second row: 50% of faculty, 50% graduate students, and 40% of under- graduate students vaccinated; third row: 100% of faculty, 100% graduate students, and 80% of undergraduate students vaccinated.

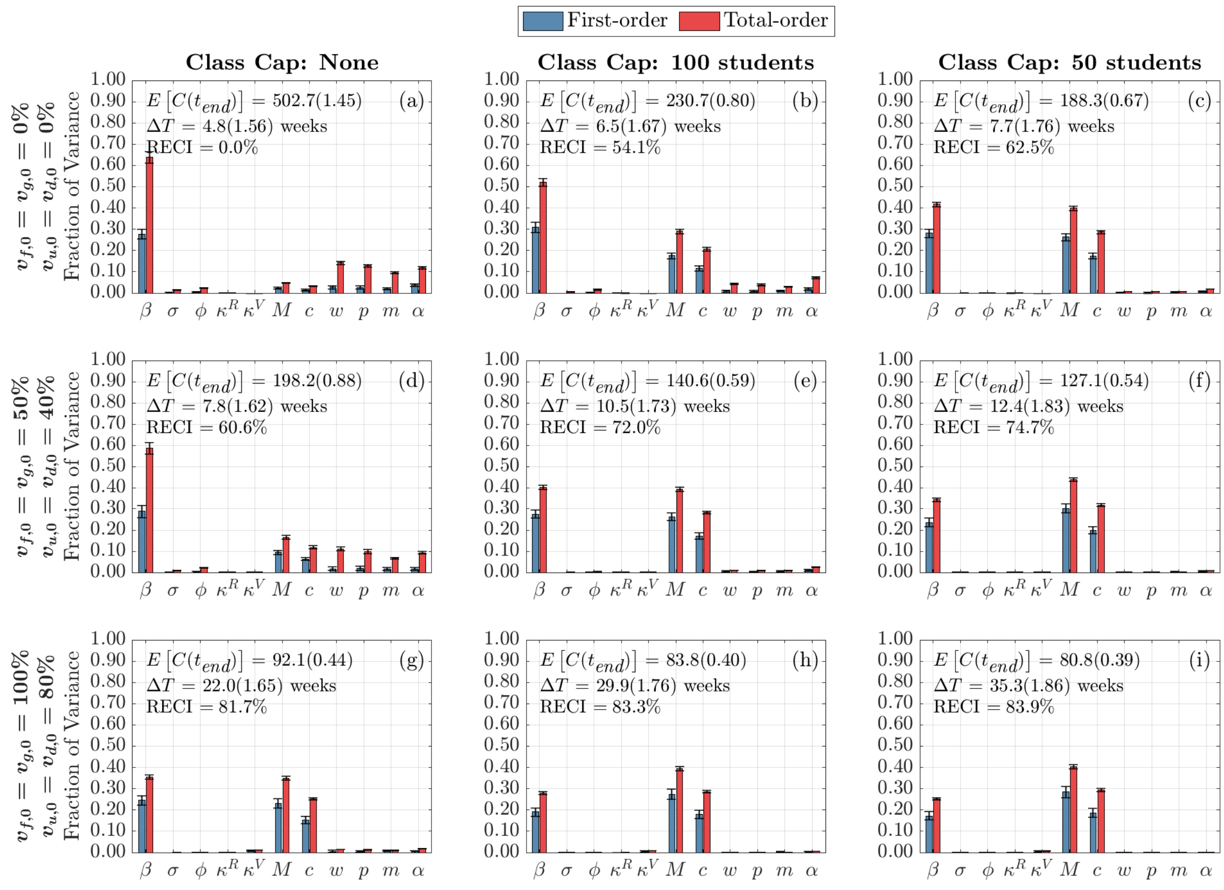

Fig. A5 Global Sensitivity Analysis of Infection and Contact Parameters on Cumulative Infections at the End of the Term. Each column represents three class cap scenarios: none, 100 student, and 50 student caps. Each row represents one of three vaccination scenarios at the start of the semester. First row: 0% vaccination; second row: 50% of faculty, 50% graduate students, and 40% of undergraduate students vaccinated; third row: 100% of faculty, 100% graduate students, and 80% of undergraduate students vaccinated.

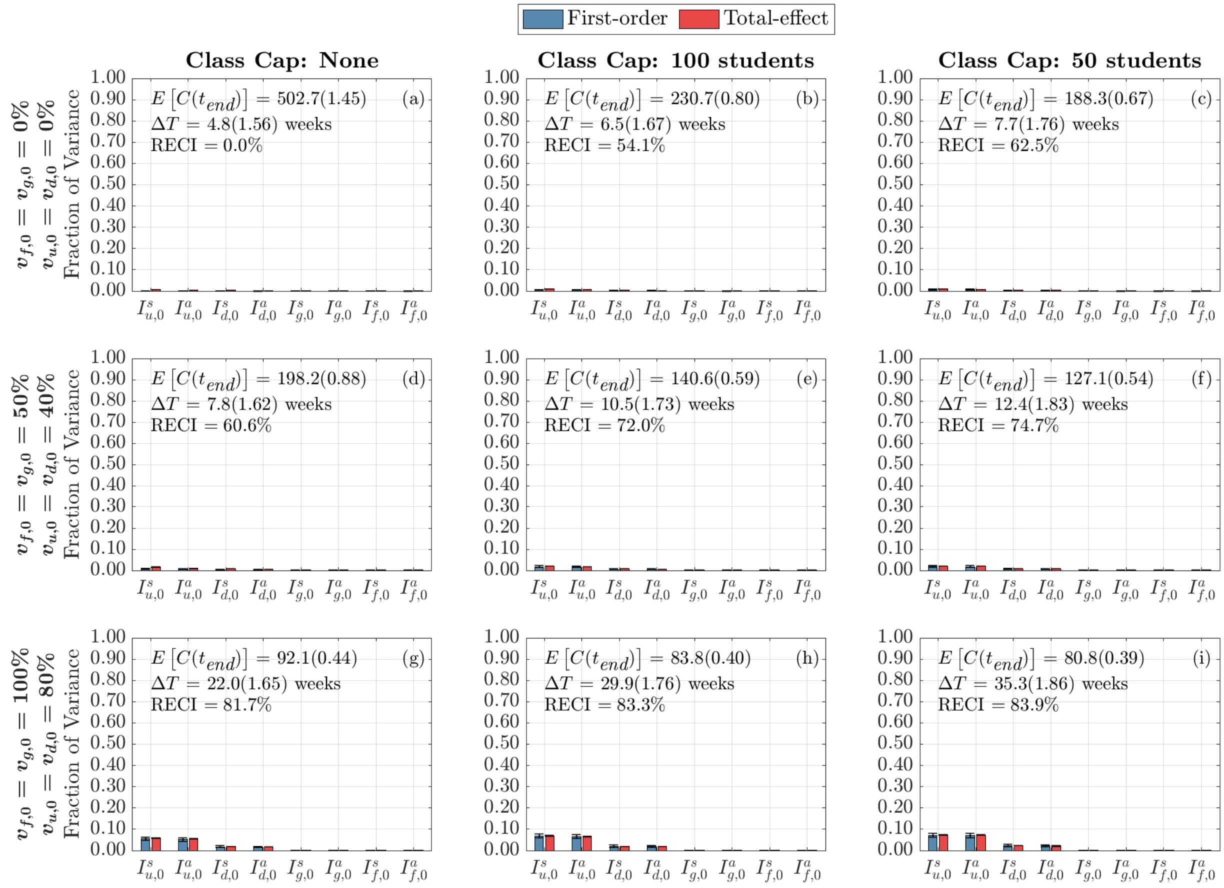

Fig. A6 Global Sensitivity Analysis of Initial Conditions on Cumulative Infections at the End of the Term. Each column represents three class cap scenarios: none, 100 student, and 50 student caps. Each row represents one of three vaccination scenarios at the start of the semester. First row: 0% vaccination; second row: 50% of faculty, 50% graduate students, and 40% of undergraduate students vaccinated; third row: 100% of faculty, 100% graduate students, and 80% of undergraduate students vaccinated.

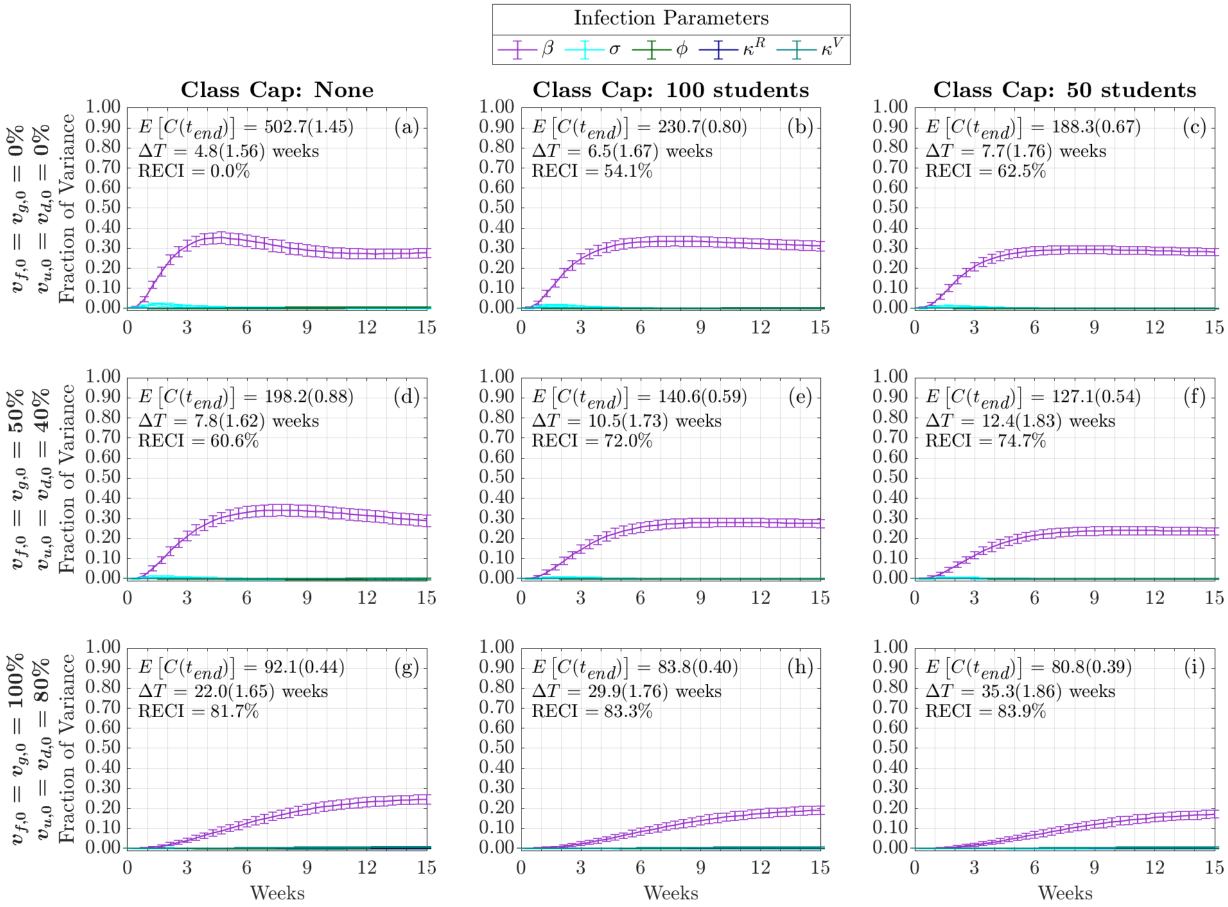

Fig. A7 Time-Varying First-Order Effect of Infection Parameters on Cumulative Infections. Each column represents three class cap scenarios: none, 100 student, and 50 student caps. Each row represents one of three vaccination scenarios at the start of the semester. First row: 0% vaccination; second row: 50% of faculty, 50% graduate students, and 40% of undergraduate students vaccinated; third row: 100% of faculty, 100% graduate students, and 80% of undergraduate students vaccinated.

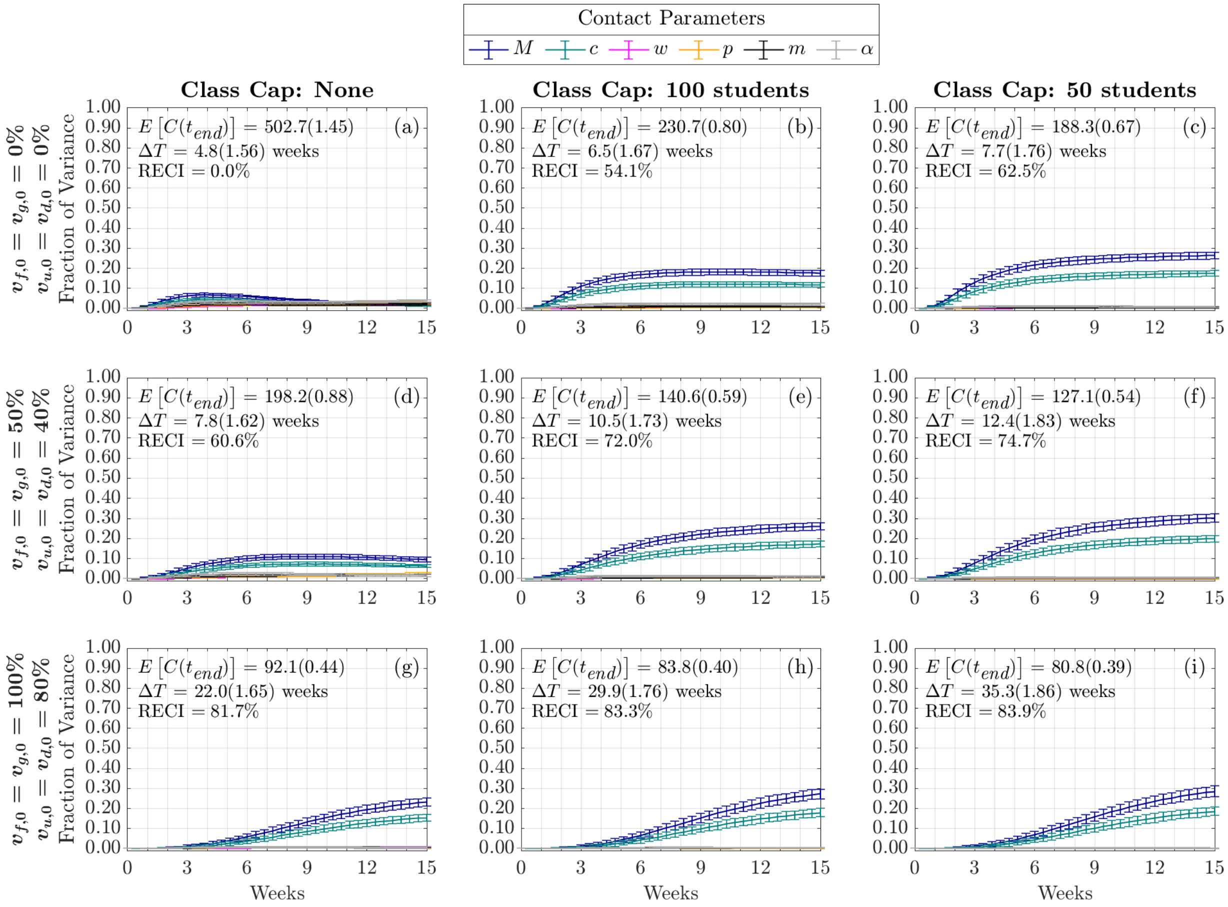

Fig. A8 Time-Varying First-Order Effect of Contact Parameters on Cumulative Infections. Each column represents three class cap scenarios: none, 100 student, and 50 student caps. Each row represents one of three vaccination scenarios at the start of the semester. First row: 0% vaccination; second row: 50% of faculty, 50% graduate students, and 40% of undergraduate students vaccinated; third row: 100% of faculty, 100% graduate students, and 80% of undergraduate students vaccinated.

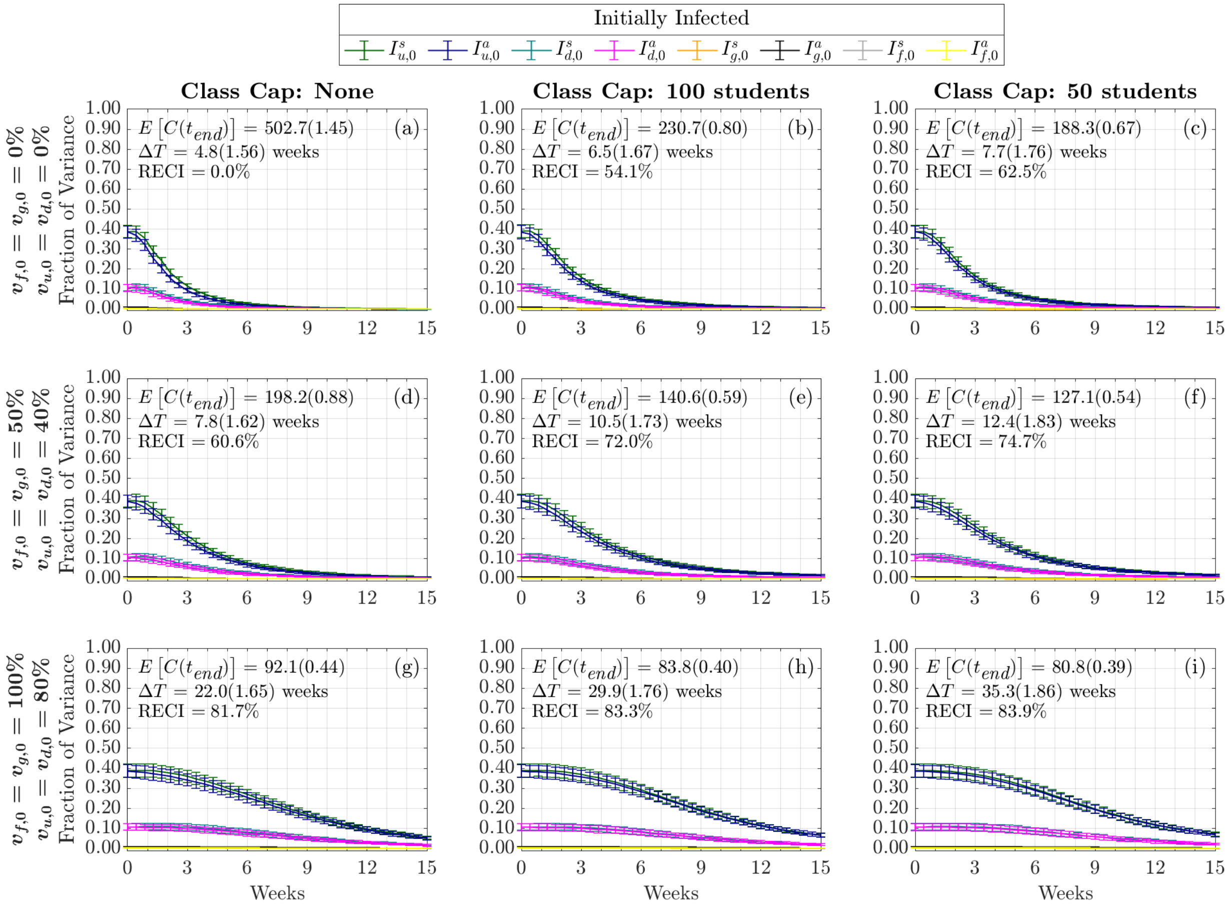

Fig. A9 Time-Varying First-Order Effect of Initial Conditions on Cumulative Infections. Each column represents three class cap scenarios: none, 100 student, and 50 student caps. Each row represents one of three vaccination scenarios at the start of the semester. First row: 0% vaccination; second row: 50% of faculty, 50% graduate students, and 40% of undergraduate students vaccinated; third row: 100% of faculty, 100% graduate students, and 80% of undergraduate students vaccinated.

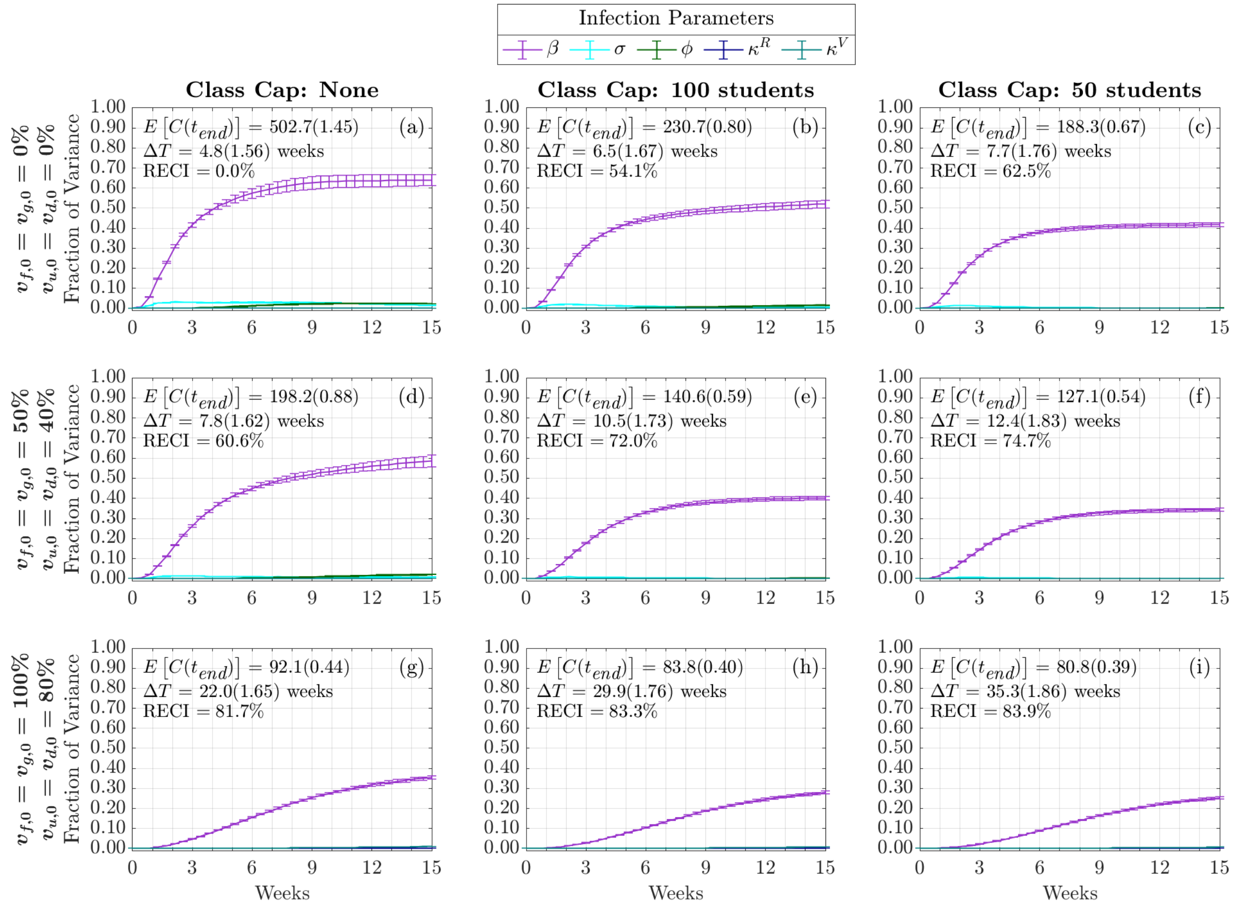

Fig. A10 Time-Varying Total-Order Effect of Infection Parameters on Cumulative Infections. Each column represents three class cap scenarios: none, 100 student, and 50 student caps. Each row represents one of three vaccination scenarios at the start of the semester. First row: 0% vaccination; second row: 50% of faculty, 50% graduate students, and 40% of undergraduate students vaccinated; third row: 100% of faculty, 100% graduate students, and 80% of undergraduate students vaccinated.

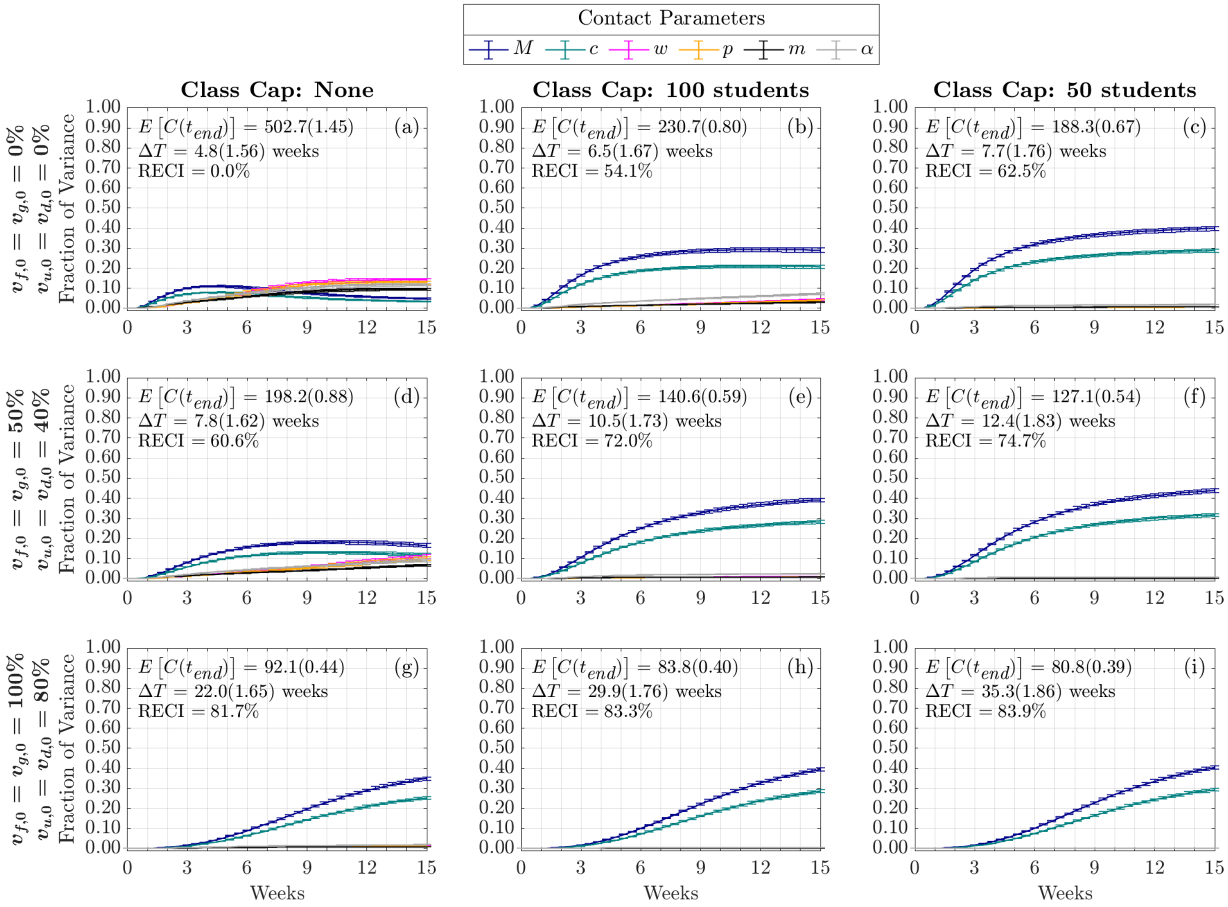

Fig. A11 Time-Varying Total-Order Effect of Contact Parameters on Cumulative Infections. Each column represents three class cap scenarios: none, 100 student, and 50 student caps. Each row represents one of three vaccination scenarios at the start of the semester. First row: 0% vaccination; second row: 50% of faculty, 50% graduate students, and 40% of undergraduate students vaccinated; third row: 100% of faculty, 100% graduate students, and 80% of undergraduate students vaccinated.

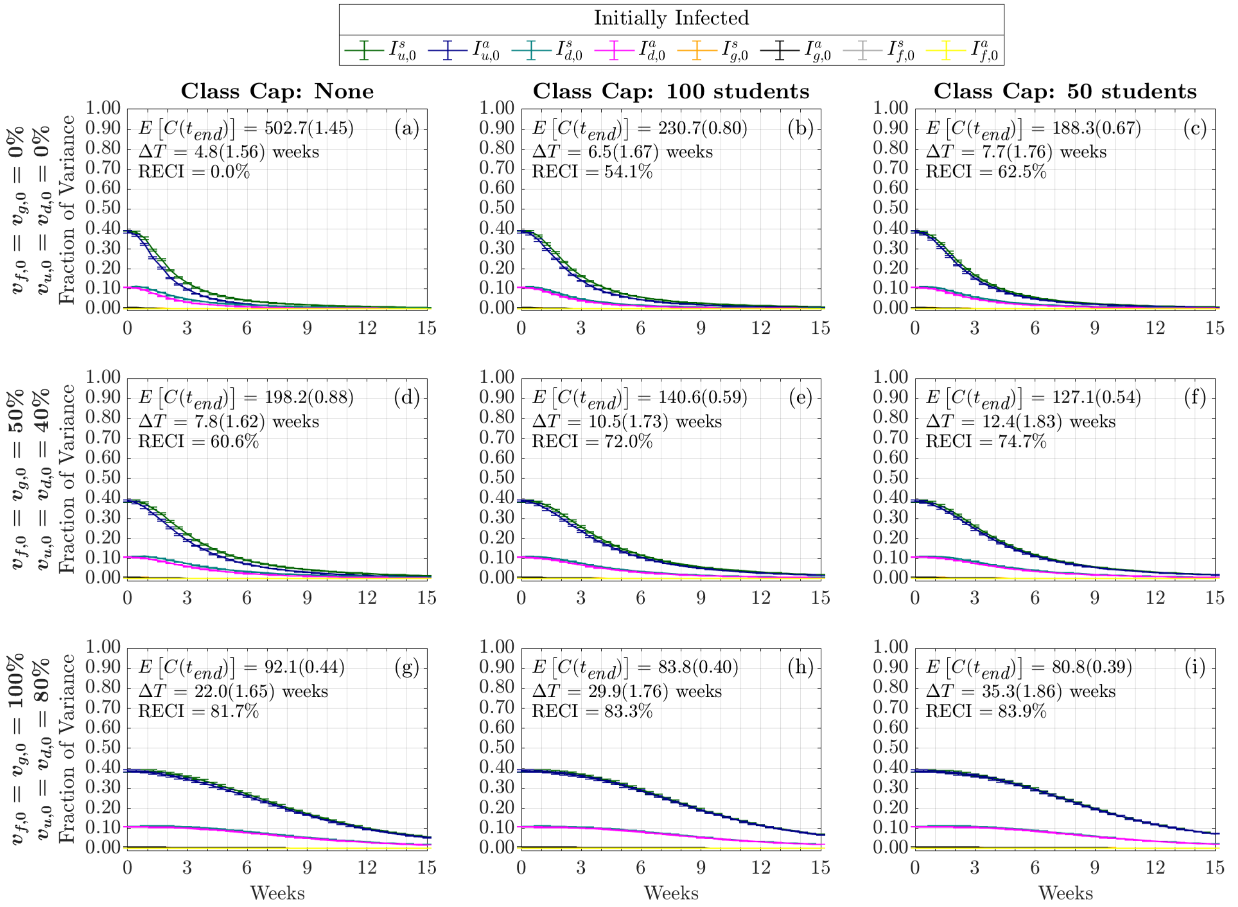

Fig. A12 Time-Varying First-Order Effect of Initial Conditions on Cumulative Infections. Each column represents three class cap scenarios: none, 100 student, and 50 student caps. Each row represents one of three vaccination scenarios at the start of the semester. First row: 0% vaccination; second row: 50% of faculty, 50% graduate students, and 40% of undergraduate students vaccinated; third row: 100% of faculty, 100% graduate students, and 80% of undergraduate students vaccinated.
