# Supplementary material for: Modeling and Global Sensitivity Analysis of Strategies to Mitigate Covid-19 Transmission on a Structured College Campus
Source: Bull Math Biol. 2023 Jan 13;85(2):13. doi: 10.1007/s11538-022-01107-2 (PMC9837465; doi:10.1007/s11538-022-01107-2)
Supplement: Supplementary file 2 — (pdf 133 KB) [file 11538_2022_1107_MOESM2_ESM.pdf]

Supplementary File 2: Modeling and Global Sensitivity Analysis of Strategies to Mitigate Covid-19 Transmission on a Structured College Campus

Lihong Zhao[*], Fabian Santiago[*], Erica M. Rutter, Shilpa Khatri, Suzanne Sindi[†]

B Network Visualization

Visualization of the university network as a weighted undirected network under four interventions with different class cap size. Edge thickness reflects weights (minimum 1 and maximum 10) **where each edge represents a classroom contact. In these figures, only classroom contacts are shown**. Nodes were colored according to their roles (reddish purple for off-campus undergraduate students, yellow for on-campus undergraduate students, sky blue for graduate students, and vermillion for faculty/staff) and sized according to weighted degree (smaller nodes correspond to smaller weighted degrees). See Table 4 for the breakdown of the networks. Those four figures were all generated using the same random seed under the same settings.

All the four figures are available at `https://github.com/slihongzhao/Supp2ZhaoBMB2022`. Note that those four figures are provided as four individual Supplementary Files in PDF format (via clickable links below) to allow the interested reader to zoom in and inspect different regions of the network. It may take a while for your PDF viewer to load due to their size.

- FigureB1_NetworkVisual_NoClassCap.pdf
  Visualization of university network as a weighted undirected network with no class caps, i.e., all classes meet in-person regardless of the number of registered students.

- FigureB2_NetworkVisual_ClassCap100.pdf
  Visualization of university network as a weighted undirected network with class cap 100, i.e., all classes with no more than 100 students registered meet in-person.

- FigureB3_NetworkVisual_ClassCap50.pdf
  Visualization of university network as a weighted undirected network with class cap 50, i.e., all classes with no more than 50 students registered meet in-person.

- FigureB4_NetworkVisual_ClassCap25.pdf
  Visualization of university network as a weighted undirected network with class cap 25, i.e., all classes with no more than 25 students registered meet in-person.

[*]These authors contributed equally to this work.
[†]Department of Applied Mathematics, University of California, Merced. ssindi@ucmerced.edu
